# Scrutinizing the datasets obtained from nanoscale features of spider silk fibres

**DOI:** 10.1038/sdata.2014.40

**Published:** 2014-10-14

**Authors:** Luciano P Silva, Elibio L Rech

**Affiliations:** 1 Embrapa Genetic Resources and Biotechnology, PBI, Parque Estação Biológica Final W5 Norte, Brasília 70770-917, DF, Brazil

**Keywords:** Atomic force microscopy, Applications of AFM, Nanoscale biophysics, Biomaterials

## Abstract

Spider silk fibres share unprecedented structural and mechanical properties which span from the macroscale to nanoscale and beyond. This is possible due to the molecular features of modular proteins termed spidroins. Thus, the investigation of the organizational scaffolds observed for spidroins in spider silk fibres is of paramount importance for reverse bioengineering. This dataset consists in describing a rational screening procedure to identify the nanoscale features of spider silk fibres. Using atomic force microscopy operated in multiple acquisition modes, we evaluated silk fibres from nine spider species. Here we present the complete results of the analyses and decrypted a number of novel features that could even rank the silk fibres according to desired mechanostructural features. This dataset will allow other researchers to select the most appropriate models for synthetic biology and also lead to better understanding of spider silk fibres extraordinary performance that is comparable to the best manmade materials.

## Background & Summary

Spider silk fibres represent the most impressive biological archetypes of high performance fibres. This stems from the fact that spider silk fibres can surprisingly merge strong tensile strength and high elasticity which are similar to those observed for steel and nylon, respectively. The last few decades have been marked by an explosion of studies describing molecular, structural, and mechanical properties of spider silk fibres^1–11^. The major reason for this interest is that spider silk fibres are one of the most promising next-generation candidates for bioinspired polymers^12^.

Among the challenges to the widespread use of spider silk fibres as models for novel materials is the understanding how spider silk proteins (spidroins) interact among them to form supramolecular nanostructures in the fibre^13,14^. This fact, together with the limited number of species represented by available molecular and mechanostructural data^15^, led to a demand for the development of methods and datasets organized to identify specific features reliable for synthetic biology approaches.

A systematic proposal toward a rational use of nanoscale features of spider silk fibres was presented in our recent Nature Communications paper^1^. There, we explored the biodiversity of spider silk fibres by several atomic force microscopy (AFM) imaging and spectroscopic modes. Using that method^1^ we and others are able to systematically select, on demand, high-performance fibres for nanoengineering purposes. However, we suggested that other researchers could experience difficulty in reproducing our experiments with exactly the same instrumental setup and conditions. Thus, we avoided reporting absolute quantities and instead relied on normalised values and ratios during comparisons among fibres in that study^1^. Here we present the datasets from that study^1^, which would be valuable to many other researchers studying the nano- and microscale mechanostructural properties of spider silk fibres.

## Methods

### Experimental design

**Figure 1:**
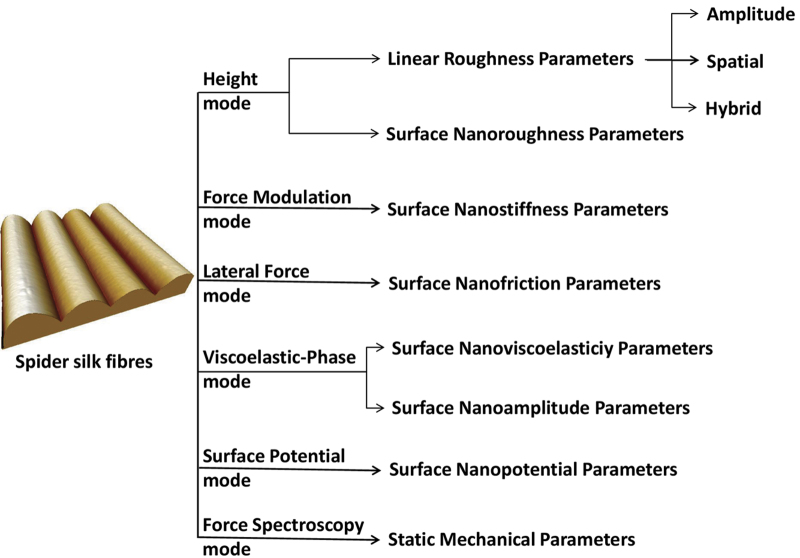
Representative scheme of the experimental design including AFM modes and corresponding quantitative parameters of the spider silk fibres.

We performed AFM analyses of natural silk fibres from nine spider species with focus on the use of multiple operation-acquisition modes under imaging and force spectroscopic approaches. A dataset of quantitative nanoscale parameters were obtained from silk fibres images and force spectroscopic data under near-native conditions (Figure 1).

### Sample preparation

Spider silks were supplied by Dr Paulo C. Motta (Institute of Biology, University of Brasilia, Brazil) under IBAMA/MMA license number (0128753 BR) and Dr Randolph V. Lewis (Utah State University, USA). Silks were reeled from adult specimens of *Aglaoctenus lagotis* (Araneomorphae, Lycosidae), *Argiope argentata* (Araneomorphae, Araneidae), *Argiope lobata* (Araneomorphae, Araneidae), *Avicularia juruensis* (Mygalomorphae, Theraphosidae), *Avicularia* sp. (Mygalomorphae, Theraphosidae), *Gasteracantha cancriformis* (Araneomorphae, Araneidae), *Nephila clavipes* (Araneomorphae, Nephilidae), *Nephilengys cruentata* (Araneomorphae, Tetragnathidae), and *Parawixia bistriata* (Araneomorphae, Araneidae). Silk fibres extruded from major ampullate gland spigots (or acinous gland spigots for *Avicularia* species) were rolled around a rotating tubular graphite at 5 mm/s, taking care to avoid contamination by silks that did not originate in the desired glands. Two synthetic silk fibres termed Recombinant-1 (MaSp-1, 16×GGLGGQGGLGGLGSQGAGLGGYGQGGAGQGGAAAAAAAAS module, a segment from a sequence previously obtained from *Parawixia bistriata*^16^ and under GenBank accession no. ADG57596.1) and Recombinant-2 (MaSp-2, 16×GPGGYGPGQQGPGGYGPGQQGPSGPGSAAAAAAAA module, a segment from a sequence previously obtained from *Nephila clavipes*^17^ and under Swiss-Prot accession no. P46804.1) were expressed, purified, and polymerised according to previously described methods^18^ and deposited on a glass coverslip. Silk fibre samples were stored in a dust-free and light-protected Petri dish under ambient conditions until analysis. Commercial nylon and steel yarns were used as controls.

### Multimode atomic force microscopy imaging analyses

AFM analyses were performed in contact, soft contact, force modulation, lateral force (trace-minus-retrace data), dynamic, phase imaging, and Kelvin force microscopy operation/acquisition modes on a commercial SPM-9600 (Shimadzu, Japan) with suitable probes^1^ in a temperature-controlled room (23 °C) under atmospheric conditions. AFM scans of similar areas were acquired at a maximum resolution of 512×512 pixels and a rate of 0.5–1 Hz. The scanned areas were perfect squares that ranged in size from 40 μm×40 μm (lower magnification) to 250 nm×250 nm (higher magnification). We used four AFM off-line software packages to qualitatively and quantitatively analyse the acquired images (SPM-9600 off-line v. 3.304—Shimadzu, Japan; SPIP v. 5.1.5—Image Metrology, Denmark; WSxM v. 5.0 Develop 3.2—Nanotec Electronica, Spain; and Gwyddion v. 2.22 for Windows—Czech Metrology Institute, Czech Republic).

All the images (including all modes) were plane-fitted automatically to compensate for any sample tilt due to misalignments of tip and fibres using SPM-9600 off-line software. Height images were also 3D-rendered using WSxM software for visual inspection and qualitative investigation. Linear roughness amplitude, spatial, and hybrid parameters were calculated along the longitudinal extension (10 μm) of five single fibres for each spider species using Gwyddion software.

Surface quantitative nanoroughness (from contact mode—height images), nanostiffness (from force modulation mode—phase images), nanofriction (from lateral force mode—horizontal deflection images), nanoviscoelasticiy (from viscoelastic mode—phase images), nanoamplitude (from viscoelastic mode—amplitude images), and nanopotential (from Kelvin force mode—phase images) were obtained using Gwyddion statistical quantities tool by measuring the mean, standard error of the mean (s.e.m.) values of each parameter; and further by calculating the relative standard error of the mean values of ten to fifteen images acquired at higher magnification (250 nm×250 nm) of each spider silk fibre for each acquisition mode.

### Single-fibre force spectroscopy analyses

Force spectroscopy experiments were performed in ambient air with the same AFM-imaged fibres. The approach-release curves were recorded at 23 °C, 30% relative humidity, and a pulling rate of 1 Hz. Twelve force-distance curves were obtained from distinct regions in five different fibres. Data for the complete sample set were acquired with the same contact or dynamic mode tips at the same AFM acquisition setup. Static nanomechanics parameters from force-distance curves were calculated using SPIP software. Maximum load, maximum pulling, and snap in forces; detachment separation and zero indentation distances; Young’ modules; and energies dissipated were calculated.

## Data Records

The dataset produced by this study have been deposited at figshare. Link to the data depositions are provided in the Data Citations section. The format, content and availability of the depositions are described in the following subsections and in Table 1.

### Data record 1—imaging and force spectroscopic data

The quantitative imaging and force spectroscopic data are contained in worksheets indicating the raw data and at the bottom of the worksheets are the calculated mean values, standard errors of the mean (s.e.m.) values, and also relative standard errors of the mean (r.s.e.m.) values (Data Citation 1). The quantitative data in figshare are organized by AFM imaging (Files 1–7), force spectroscopic (File 8) modes and AFM probes technical data (File 9). The column details are given bellow.

### File 1. linear roughness parameters.xlsx

This data file carries three worksheets containing raw data and descriptive statistics regarding roughness (amplitude, spatial and hybrid) parameters obtained in the longitudinal direction of the spider silk fibres using Gwyddion software (Windows, v. 2.22). These parameters are described in the English version of Gwyddion user guide available on-line at http://gwyddion.net/documentation/user-guide-en/ (pages 60–61) and also shared in the slides of a developers talk available on-line at http://gwyddion.net/presentations/talk-roughness-David-Necas-2012.pdf.

The column descriptions for the raw data worksheets are as follows:

#### Amplitude parameters

COLUMN A—Sample Label.

COLUMN B—Roughness Average (Ra)—Arithmetical mean deviation. The average deviation of all points roughness profile from a mean line over the evaluation length.

COLUMN C—Root mean square roughness (Rq)—The average of the measured height deviations taken within the evaluation length and measured from the mean line.

COLUMN D—Maximum height of the roughness (Rt)—Maximum peak-to-peak-valley height. The absolute value between the highest and lowest peaks.

COLUMN E—Maximum roughness valley depth (Rv)—Lowest valley. There is the depth of the deepest valley in the roughness profile over the evaluation length.

COLUMN F—Maximum roughness peak height (Rp)—Highest peak. There is the height of the highest peak in the roughness profile over the evaluation length.

COLUMN G—Average maximum height of the roughness (Rtm)—Mean peak-to-valley roughness. It is determined by the difference between the highest peak ant the lowest valley within multiple samples in the evaluation length.

COLUMN H—Average maximum roughness valley depth (Rvm)—The mean valley depth based on one peak per sampling length. The single deepest valley is found in five sampling lengths (m=5) and then averaged.

COLUMN I—Average maximum roughness peak height (Rpm)—The mean peak height based on one peak per sampling length. The single highest peak is found in five sampling lengths (m=5) and then averaged.

COLUMN J—Average third highest peak to third lowest valley height (R3z)—The distance between the third highest peak and the third lowest valley. A peak is a portion of the surface above the mean line crossings.

COLUMN K—Average third highest peak to third lowest valley height (R3z ISO)—The height of the third highest peak from the third lowest valley per sampling length. The base roughness depth is found in five sampling lengths and then averaged.

COLUMN L—Average maximum height of the profile (Rz)—The average absolute value of the five highest peaks and the five lowest valleys over the evaluation length.

COLUMN M—Average maximum height of the roughness (Rz ISO)—The average peak-to-valley roughness based on one peak and one valley per sampling length. The single largest deviation is found in five sampling lengths and then averaged.

COLUMN N—Skewness (Rsk)—Skewness is a parameter that describes the shape of the amplitude distribution function (ADF) that is the probability function that gives the probability that a profile of the surface has a certain height *z* at any position *x*. Skewness is a simple measure of the asymmetry of the ADF, or, equivalently, it measures the symmetry of the variation of a profile about its mean line.

COLUMN O—Kurtosis (Rku)—Kurtosis is the ADF shape parameter considered. Kurtosis relates to the uniformity of the ADF or, equivalently, to the spikiness of the profile.

COLUMN P—Waviness average (Wa)—A typically used number to describe waviness of the surface and that is closely related to the Ra.

COLUMN Q—Root mean square waviness (Wq)—The average of the measured waviness value deviations.

COLUMN R—Waviness maximum height (Wy=Wmax)—The difference value between the highest point and the lowest point of a measurement curve.

COLUMN S—Maximum height of the profile (Pt)—The maximum peak to valley height of the profile in the assessment length.

#### Spatial parameters

COLUMN A—Sample Label.

COLUMN B—Average wavelength of the profile (lambda a)—The average wavelength that can be calculated from average roughness (nm) and average absolute slope (degree).

COLUMN C—Root mean square (RMS) wavelength of the profile (lambda q)—The average of the measured wavelength of the profile deviations.

#### Hybrid parameters

COLUMN A—Sample Label

COLUMN B—Average absolute slope (delta a)—The average absolute slope of the profile.

COLUMN C—Root mean square (RMS) slope (delta q) (10^−6^)—The average absolute slope of the profile deviations.

COLUMN D—Length (L)—The length of the profile.

COLUMN E—Developed profile length (L0)

COLUMN F—Profile length ratio (Lr)

### File 2. surface nanoroughness parameters.xlsx

This data file carries one worksheet containing raw data and descriptive statistics regarding surface roughness parameters obtained from square areas of height images (dynamic mode) of the spider silk fibres using Gwyddion software (Windows, v. 2.22). These parameters are described in the English version of Gwyddion user guide available on-line at http://gwyddion.net/documentation/user-guide-en/ (pages 53–54) and also shared in the slides of a developers talk available on-line at http://gwyddion.net/presentations/talk-roughness-David-Necas-2012.pdf.

The column descriptions for the raw data worksheet are as follows:

COLUMN A—Sample Label

COLUMN B—Average Value

COLUMN C—Minimum

COLUMN D—Maximum

COLUMN E—Median

COLUMN F—Ra

COLUMN G—Rms

COLUMN H—Skewness

COLUMN I—Kurtosis

COLUMN J—Surface Area

COLUMN K—Projected Area

COLUMN L—Inclination (theta)

COLUMN M—Inclination (phi).

### File 3. surface nanostiffness parameters.xlsx

This data file carries one worksheet containing raw data and descriptive statistics regarding surface stiffness parameters obtained from square areas of phase images (force modulation mode) of the spider silk fibres using Gwyddion software (Windows, v. 2.22). These parameters are described in the English version of Gwyddion user guide available on-line at http://gwyddion.net/documentation/user-guide-en/ (pages 53–54) and also shared in the slides of a developers talk available on-line at http://gwyddion.net/presentations/talk-roughness-David-Necas-2012.pdf.

The column descriptions for the raw data worksheet are as follows:

COLUMN A—Sample Label

COLUMN B—Average Value

COLUMN C—Minimum

COLUMN D—Maximum

COLUMN E—Median

COLUMN F—Ra

COLUMN G—Rms

COLUMN H—Skewness

COLUMN I—Kurtosis.

### File 4. surface nanofriction parameters.xlsx

This data file contains one worksheet containing raw data and descriptive statistics regarding surface frictional parameters obtained from square areas of horizontal deflection images (lateral force mode) of the spider silk fibres using Gwyddion software (Windows, v. 2.22). These parameters are described in the English version of Gwyddion user guide available on-line at http://gwyddion.net/documentation/user-guide-en/ (pages 53–54) and also shared in the slides of a developers talk available on-line at http://gwyddion.net/presentations/talk-roughness-David-Necas-2012.pdf.

The column descriptions for the raw data worksheet are as follows:

COLUMN A—Sample Label

COLUMN B—Average Value

COLUMN C—Minimum

COLUMN D—Maximum

COLUMN E—Median

COLUMN F—Ra

COLUMN G—Rms.

### File 5. surface nanoviscoelasticiy parameters.xlsx

This data file contains one worksheet containing raw data and descriptive statistics regarding surface viscoelastic parameters obtained from square areas of phase images (viscoelastic-phase mode) of the spider silk fibres using Gwyddion software (Windows, v. 2.22). These parameters are described in the English version of Gwyddion user guide available on-line at http://gwyddion.net/documentation/user-guide-en/ (pages 53–54) and also shared in the slides of a developers talk available on-line at http://gwyddion.net/presentations/talk-roughness-David-Necas-2012.pdf.

The column descriptions for the raw data worksheet are as follows:

COLUMN A—Sample Label

COLUMN B—Average Value

COLUMN C—Minimum

COLUMN D—Maximum

COLUMN E—Median

COLUMN F—Ra

COLUMN G—Rms

COLUMN H—Skewness

COLUMN I—Kurtosis.

### File 6. surface nanoamplitude parameters.xlsx

This data file contains one worksheet containing raw data and descriptive statistics regarding surface stiffness parameters obtained from square areas of amplitude images (viscoelastic-phase mode) of the spider silk fibres using Gwyddion software (Windows, v. 2.22). These parameters are described in the English version of Gwyddion user guide available on-line at http://gwyddion.net/documentation/user-guide-en/ (pages 53–54) and also shared in the slides of a developers talk available on-line at http://gwyddion.net/presentations/talk-roughness-David-Necas-2012.pdf.

The column descriptions for the raw data worksheet are as follows:

COLUMN A—Sample Label

COLUMN B—Average Value

COLUMN C—Minimum

COLUMN D—Maximum

COLUMN E—Median

COLUMN F—Ra

COLUMN G—Rms

COLUMN H—Skewness

COLUMN I—Kurtosis.

### File 7. surface nanopotential parameters.xlsx

This data file contains one worksheet containing raw data and descriptive statistics regarding surface potential parameters obtained from square areas of Kelvin force microscopy images (surface potential mode) of the spider silk fibres using Gwyddion software (Windows, v. 2.22). These parameters are described in the English version of Gwyddion user guide available on-line at http://gwyddion.net/documentation/user-guide-en/ (pages 53–54) and also shared in the slides of a developers talk available on-line at http://gwyddion.net/presentations/talk-roughness-David-Necas-2012.pdf.

The column descriptions for the raw data worksheet are as follows:

COLUMN A—Sample Label

COLUMN B—Ra

COLUMN C—Rms.

### File 8. static mechanical parameters.xlsx

This data file contains one worksheet containing raw data and descriptive statistics regarding static mechanical parameters obtained from force-distance curves of the spider silk fibres using SPIP software and are described in its manual.

The column descriptions for the raw data worksheet are as follows:

COLUMN A—Sample Label.

COLUMN B—Maximum Load Force (Max Ld)—The maximum loading force usually at the left end of the approach curve.

COLUMN C—Maximum Pulling Force (Max Pull)—The maximum pulling force on the retraction curve.

COLUMN D—Snap In (Snap In)—The magnitude of the force immediately after snap in.

COLUMN E—Detachment Separation (Detach Sep)—The separation (distance between tip apex and the maximum indentation point) at detachment.

COLUMN F—Young’s Modulus (Youngs Mod)—The elastic modulus calculated by one of the indentation fitting models on the Force versus Separation curve.

COLUMN G—Zero Indentation (Zero Ind)—The point in the Force versus Separation graph which is used as zero point for indentation fitting.

COLUMN H—Dissipated Energy (Energy)—The area between the approach curve and the retraction curve of the force versus separation curve.

### File 9. AFM probes technical data.doc

This data file contains one table with the description of the AFM probes used to obtain the images and force curve spectra of the spider silk fibres.

COLUMN 1—AFM operation/acquisition mode.

COLUMN 2—Cantilever type.

COLUMN 3—Cantilever metal coating.

COLUMN 4—Cantilever length.

COLUMN 5—Cantilever resonant frequency.

COLUMN 6—Cantilever spring constant.

COLUMN 7—Tip type.

COLUMN 8—Tip curvature radius.

COLUMN 9—Tip material.

COLUMN 10—Probe manufacturer/model.

### File 10. linear roughness parameters.zip

This data file contains raw data AFM height mode images in ASCII format and which were used for obtain linear roughness parameters.

### File 11. surface roughness parameters.zip

This data file contains raw data AFM height mode images in Shimadzu proprietary format and which were used for obtain surface roughness parameters.

### File 12. surface nanostiffness parameters.zip

This data file contains raw data AFM force modulation mode (phase) images in Shimadzu proprietary format and which were used for obtain surface nanostiffness parameters.

### File 13. surface nanofriction parameters.zip

This data file contains raw data AFM lateral force mode (horizontal deflection—trace and retrace) images in Shimadzu proprietary format and which were used for obtain surface nanofriction parameters.

### File 14. surface nanoviscoelasticity parameters.zip

This data file contains raw data AFM viscoelastic mode (phase) images in Shimadzu proprietary format and which were used for obtain surface nanoviscoelasticity parameters.

### File 15. surface nanoamplitude parameters.zip

This data file contains raw data AFM viscoelastic mode (amplitude) images in Shimadzu proprietary format and which were used for obtain surface nanoamplitude parameters.

### File 16. surface nanopotential parameters.zip

This data file contains raw data AFM Kelvin probe mode (potential) images in Shimadzu proprietary format and which were used for obtain surface nanopotential parameters.

### File 17. static mechanical parameters.zip

This data file contains raw data AFM force spectroscopy mode (force curves) in ASCII format and which were used for obtain static mechanical parameters.

## Technical Validation

### Validation of the study

The experimental design presented in this dataset has been validated in several ways. Firstly, forward (trace) and backward (retrace) scans were performed for all imaging modes as well as approach and retraction curves were performed for all force spectroscopic modes both to ensure that no distortion was occurring at any level. Secondly, the same AFM tip was used when direct comparisons were required to ensure similar tip geometry and cantilever spring constant. Finally, it has been largely demonstrated that spider silk fibres show high degree of variability in their structural and mechanical properties at interspecific, intraspecific and intraindividual levels^15^. Thus, absolute values of the present data descriptor must be taken with careful consideration.

## Usage Notes

There are several predictable uses for these datasets. Firstly, they suggest candidate spider species and consequently their silk fibres for further molecular studies based on nanoscale characteristics. Secondly, it could be used to select which fibres display desired mechanostructural features. Lastly, these datasets can be used to compare with nanoscale features of several other materials, including synthetic fibres, plastics, metals, ceramics, glasses, papers, woods, and resins.

## Additional information

**How to cite this article:** Silva, L. P. and Rech, E. L. Scrutinizing the datasets obtained from nanoscale features of spider silk fibres. *Sci. Data* 1:140040 doi: 10.1038/sdata.2014.40 (2014).

## Supplementary Material



## Figures and Tables

**Table 1:** Availability of measured AFM data as deposited on FigShare

**AFM mode**	**Measured parameters**	**Tabulated data (worksheets)**	**Raw data (images and force curves)**
Contact/Dynamic modes (height)	Linear roughness	All	All
Contact/Dynamic modes (height)	Surface roughness	All	All
Force modulation mode (phase)	Surface nanostiffness	All	Representative for 4 species
Lateral force mode (deflection)	Surface nanofriction	All	Representative for 7 species
Viscoelastic mode (phase)	Surface nanoviscoelasticity	All	All
Viscoelastic mode (amplitude)	Surface nanoamplitude	All	All
Kelvin probe mode (potential)	Surface nanopotential	All	Representative
Force-distance curves (contact)	Static Mechanical	All	Representative for 8 species and manmade materials
(Data Citation 1).			
